# A Combination of Activation and Repression by a Colinear Hox Code Controls Forelimb-Restricted Expression of *Tbx5* and Reveals Hox Protein Specificity

**DOI:** 10.1371/journal.pgen.1004245

**Published:** 2014-03-20

**Authors:** Satoko Nishimoto, Carolina Minguillon, Sophie Wood, Malcolm P. O. Logan

**Affiliations:** 1 Division of Developmental Biology, MRC-National Institute for Medical Research, Mill Hill, London, United Kingdom; 2 Randall Division of Cell and Molecular Biophysics, King's College London, London, United Kingdom; 3 CSIC-Institut de Biologia Molecular de Barcelona, Parc Científic de Barcelona, Barcelona, Spain; 4 Procedural Services Section, MRC-National Institute for Medical Research, Mill Hill, London, United Kingdom; University of Geneva, Switzerland

## Abstract

Tight control over gene expression is essential for precision in embryonic development and acquisition of the regulatory elements responsible is the predominant driver for evolution of new structures. *Tbx*5 and *Tbx4*, two genes expressed in forelimb and hindlimb-forming regions respectively, play crucial roles in the initiation of limb outgrowth. Evolution of regulatory elements that activate *Tbx5* in rostral LPM was essential for the acquisition of forelimbs in vertebrates. We identified such a regulatory element for *Tbx5* and demonstrated Hox genes are essential, direct regulators. While the importance of Hox genes in regulating embryonic development is clear, Hox targets and the ways in which each protein executes its specific function are not known. We reveal how nested Hox expression along the rostro-caudal axis restricts *Tbx5* expression to forelimb. We demonstrate that Hoxc9, which is expressed in caudal LPM where *Tbx5* is not expressed, can form a repressive complex on the *Tbx5* forelimb regulatory element. This repressive capacity is limited to Hox proteins expressed in caudal LPM and carried out by two separate protein domains in Hoxc9. Forelimb-restricted expression of *Tbx5* and ultimately forelimb formation is therefore achieved through co-option of two characteristics of Hox genes; their colinear expression along the body axis and the functional specificity of different paralogs. Active complexes can be formed by Hox PG proteins present throughout the rostral-caudal LPM while restriction of *Tbx5* expression is achieved by superimposing a dominant repressive (Hoxc9) complex that determines the caudal boundary of *Tbx5* expression. Our results reveal the regulatory mechanism that ensures emergence of the forelimbs at the correct position along the body. Acquisition of this regulatory element would have been critical for the evolution of limbs in vertebrates and modulation of the factors we have identified can be molecular drivers of the diversity in limb morphology.

## Author Summary

The acquisition of limbs during vertebrate evolution was a very successful innovation that enabled this group of species to diversify and colonise land. It has become clear recently that the primary driver behind the evolution of new structures, such as limbs, is the acquisition of novel regulatory elements that control when and where genes are activated rather than the proteins encoded by the genes themselves acquiring novel functions. We have identified the regulatory element from a gene, *Tbx5*. Activation of *Tbx5* in the forelimb-forming region of the developing embryos is essential for forelimbs to form and disruption of human *TBX5* causes limb abnormalities. We show that activation of *Tbx5* in a restricted territory is achieved through a combination of activation inputs that are present broadly throughout the embryo flank and dominant, repressive inputs present only in more caudal regions of the flank. The sum of these inputs yields restricted activation in the rostral, forelimb-forming flank. Our results explain how the regulatory switches that were harnessed for the acquisition of limbs during evolution operate and how they can be turned off during the evolution of limblessness in species such as the snake.

## Introduction

Forelimbs and hindlimbs are derivatives of the lateral plate mesoderm (LPM) that arise at fixed positions along the vertebrate body axis. Limb formation is initiated by limb induction signals from axial tissues [1]. The presumptive limb-forming regions initially express two T-box genes prior to overt limb bud formation, *Tbx5* in nascent forelimbs and *Tbx4* in hindlimbs [2]–[5]. Genetic studies in the mouse have shown that both genes are crucial for normal limb outgrowth by activating *Fgf10* in the limb mesenchyme [6]–[8]. Fgf10 subsequently induces *Fgf8* expression in the apical ectodermal ridge (AER) and Fgf8 produced from the AER, in turn, maintains *Fgf10* expression in mesenchyme to establish a positive feedback loop of Fgf signalling that maintains limb growth. Mutations in human *TBX5* cause Holt-Oram Syndrome (HOS OMIM142900), a disorder characterised by upper limb and heart abnormalities [9], [10] and mutations in *TBX4* cause Small Patella Syndrome (SPS OMIM 147891), a disorder characterised by knee, pelvis and toe defects [11]. *Tbx5* is the earliest marker of presumptive forelimb mesenchyme and because activation of this factor within a defined region of the LPM ultimately dictates the position at which the forelimbs will arise, identifying the factors that control activation of this *Tbx5* expression domain will reveal the mechanisms employed that allowed the acquisition of limbs in vertebrates and that dictate forelimb position in the embryo.

*Tbx5* is initially expressed in the forelimb-forming region of LPM prior to the emergence of a bud and it is subsequently restricted to the forelimb region as development proceeds. *Tbx5* is essential for forelimb formation and this exclusive requirement is limited to a short time window when limb bud initiation occurs [12]. *Tbx4*, the paralog of *Tbx5*, is able to rescue forelimb formation following conditional deletion of *Tbx5* [13]. Furthermore, the ancestral *Tbx4/5* gene represented by *AmphiTbx4/5* of the limbless cephalochordate, amphioxus, can fully compensate for the loss of *Tbx5* in the mouse [14]. This indicates the ancestral protein from a limbless organism has limb-inducing potential and supports a model in which evolution of a regulatory element sufficient to activate *Tbx5* expression in the LPM was a critical step in the acquisition of limbs during vertebrate evolution.

Hox genes are conserved homeodomain-containing transcription factors that are arranged in clusters in the genome. The chromosomal organization of the genes in the complex reflects their expression pattern along the rostro-caudal body axis to determine positional identity [15], [16]. As relative positions of limbs, axial vertebrae and Hox expression domains are conserved among vertebrates in spite of the variable numbers of each type of vertebrae (e.g. cervical, thoracic, lumbar and sacral vertebrae), Hox genes have been good candidates as determinants of limb position [17], [18]. Despite the unquestionable importance of Hox genes in patterning the developing embryo, very little is known about their direct targets and mechanisms of action.

We have previously identified a *Tbx5* regulatory element sufficient for early forelimb expression [19]. This element contains Hox binding sites that are required for the enhancer activity, thus implicating Hox genes in direct, positive regulation of *Tbx5*. However, since the ability to activate *Tbx5* is not strictly restricted to Hox genes expressed only at forelimb level, the mechanism by which a rostro-caudal Hox code establishes forelimb-restriction of *Tbx5* remained unknown. Here, we demonstrate how Hox paralogous group members act cooperatively to restrict expression of *Tbx5* in the LPM, which ultimately determines the positions the forelimbs will emerge from the flank of the embryo. We show that mutations of a single Hox binding site in the *Tbx5* forelimb regulatory element cause expanded reporter gene expression in caudal LPM. Rostral restriction in *Tbx5* expression through repression in the caudal LPM is mediated by Hoxc8/9/10 genes and this repressive function is limited to Hox genes that are expressed in *Tbx5*-negative caudal LPM. We further map the Hoxc9 protein domains required to confer transcriptional repression that distinguishes these paralogs from other Hox proteins expressed throughout the flank of the embryo. Our results demonstrate how a nested, combinatorial code of Hox protein transcriptional activation and repression along the rostro-caudal embryo axis restricts *Tbx5* expression to the forelimb and ultimately determines forelimb position.

## Results

### Hox binding sites are required for forelimb-restricted *Tbx5* expression

**Figure 1:**
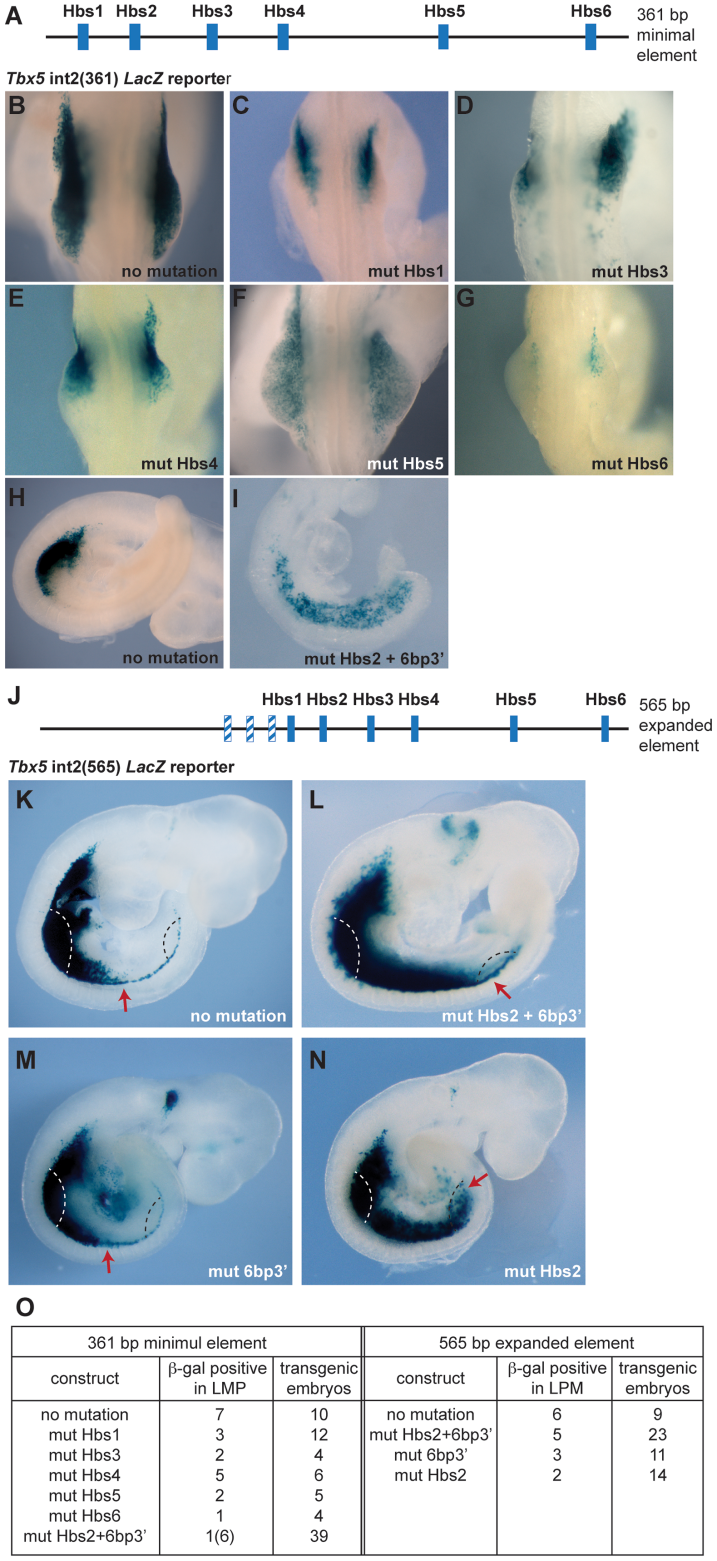
Mutation analysis of the *Tbx5* forelimb regulatory element. **A.** Schematic representation of the mouse *Tbx5* forelimb regulatory element. This 361 bp sequence contains six Hox binding sites (Hbs; blue boxes). **B–I.** E9.5 trangenic embryos (B–H) and E9.0 embryo (I) stained for β-galactosidase. Control (B and H) and mutated constructs (C–G and I) of the *Tbx5* int2(361) reporter. **C–G and I** Results following mutation of one of the six Hox binding sites; mutation of Hbs1 (C), Hbs3 (D), Hbs4 (E), Hbs5 (F), Hbs6 (G) or Hbs2 and an additional 6 bp sequence located 3′ of Hbs2 (6bp3′) (I) **J.** Schematic representation of the 565 bp fragment of the *Tbx5* regulatory element. The additional 204 bp sequence contains three putative Hox binding sites (blue/white hatched boxes). **K–N.** Representative β-galactosidase stainings for WT *Tbx5* int2(565) reporter construct (K), construct with mutation(s) on both Hbs2 and 6bp3′ (L), 6bp3′ (M) or Hbs2 (N) alone. The red arrows indicate the caudal extent of staining. Forelimb bud (white dashed line) and presumptive hindlimb region (black dashed line) were marked. **O.** Tabulation of the number of embryos showing *lacZ* expression.

Previously, we identified a short regulatory element within intron 2 of the mouse *Tbx5* gene that recapitulates the dramatic forelimb-restricted expression of this gene [19]. This 361 base pair (bp) sequence contains six Hox binding sites (Hbs) (Fig. 1A). To analyze which sites within this minimal element are required for *Tbx5* expression, we generated a series of constructs in which each individual Hbs site1-6 is mutated and tested their ability to activate a *LacZ* reporter gene in transgenic mice. While the *Tbx5* int2(361) reporter construct drove forelimb-restricted expression of *LacZ* (Fig. 1B, H and [19]), mutation of either individual Hbs1, or Hbs3, or Hbs4, or Hbs5, or Hbs6 resulted in reduced reporter gene expression (Fig. 1C–G). Interestingly, in most cases residual expression was consistently detected in the anterior forelimb bud, however, mutation of Hbs5 produced mosaic expression throughout the limb. The six bp sequence (TGAGAG, bottom strand) situated 3′ of Hbs2 (6bp3′) is similar but not identical to both Pbx (TGAT) and Meis (TGACAG) canonical binding sequences [20]. Pbx and Meis are Hox cofactors that can bind DNA as heterodimers. Mutation of Hbs2 and 6bp3′ in the 361 bp core fragment produced a strikingly different result. Reporter expression was detected in the forelimb but was now also expanded throughout the interlimb and hindlimb-forming region (Fig. 1I). Since the number of transgenic embryos that showed expression with this construct was low, to study the effect of mutating the individual sites further, we used a 565 bp fragment (*Tbx5* int2(565)) (Fig. 1J) that contains an additional 204 bp sequence 5′ to the 361 bp core element. The extra 204 bp sequence contains three putative Hox binding sites. However, these sites are not required to control the spatial restriction of expression since the 361 bp fragment produces forelimb-restricted expression equivalent to that observed with the 565 bp fragment (Fig. 1K and [19]). We have also previously shown that the fragment containing this 204 bp sequence and Hbs1 and Hbs2 cannot activate reporter gene expression indicating these sites are not sufficient for the enhancer activity [19]. As observed with the smaller fragment, mutations of Hbs2 and 6bp3′ caused caudally expanded *LacZ* reporter activity to include the LPM of interlimb and hindlimb-forming regions, which never normally express *Tbx5* (Fig. 1L). These results suggest that these sites are required to restrict *Tbx5* expression to the forelimb-forming region. The activity of the Hbs2+6bp3′-mutated construct is dependent on the presence of the other Hox sites since mutation of Hbs2 and 6bp3′ together with Hbs3-6 did not drive reporter expression at all (n = 0/6, data not shown). To distinguish the requirement for Hbs2 and 6bp3′, we next mutated either of these sites (Fig. 1M–N). Mutation of 6bp3′ did not affect the expression domain of the *LacZ* reporter (Fig. 1M), while mutation of Hbs2 caused caudal expansion (Fig. 1N) equivalent to that seen after mutating both Hbs2 and 6bp3′ (Fig. 1L). These results suggest that Hbs2 plays the predominant role restricting *Tbx5* expression to the forelimb-forming region.

These results demonstrate that the binding sites within this regulatory element can be divided into 2 distinct functional groups. Hbs1 and 3-6 act as ‘on’ switches important for the amplitude of activation, whereas Hbs2 determines spatial resolution by hosting repressive complexes that restrict the domain of activation. This element can therefore have a binary function, serving as a site for the formation of transcriptional activation or repression complexes.

### *Hoxc8*, *Hoxc9* and *Hoxc10* genes are expressed in *Tbx5*-negative, caudal lateral plate mesoderm

**Figure 2:**
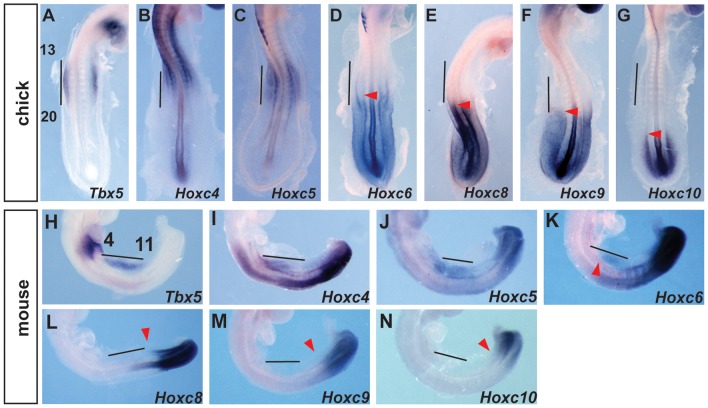
Comparison of *Tbx5* and *HoxC* gene expression domains. **A–G.** *In situ* hybridization for *Tbx5* (A) or *HoxC* genes (B–G) on 20–22 somites stages chick embryos. *Tbx5* is expressed lateral to somites 13–20. The forelimb-forming region is indicated by the vertical bar. The red arrowheads indicate the rostral extent of expression. **H–N.** *In situ* hybridization for *Tbx5* (H) or *HoxC* (I–N) on 11–13 somite stages mouse embryos. *Tbx5* is expressed lateral to somites 4–11. The forelimb-forming region is indicated by the vertical bar. The red arrowheads indicate the rostral extent of expression.

The presence of Hox binding sites in this element prompted us to search for candidate Hox genes that could be acting on this element as either positive or negative regulators of transcription. Previously, we have shown that PG 4 and 5 *Hox* genes can activate this regulatory element [19]. We now focused on Hox factors that could be mediating spatial resolution of this regulatory element by forming repressive complexes. We analysed the expression of *Hox* genes in chick and mouse embryos at stages when *Tbx5* is first expressed in the forelimb-forming region. *Tbx5* is first expressed at the level of somites 13–20 in chick and somites 4–11 in mouse embryos. As previously reported [19] the expression domains of *Hox4* and *Hox5* paralogs overlap with that of *Tbx5* in both mouse and chick embryos (Fig. 2A–C, H–J). Since the expression patterns of *HoxA*, *HoxB*, *HoxC* and *HoxD* cluster genes are broadly similar, we show here the results of the *HoxC* cluster genes, as a representative example for simplicity. *Hoxc6* is expressed within the caudal-most domain of *Tbx5* as well as in more caudal LPM (Fig. 2D and K). Conversely, *Hoxc8*, *Hoxc9* and *Hoxc10* are exclusively expressed in caudal domains of the LPM that do not express *Tbx5* (Fig. 2E–G and L–N) and are therefore candidates to repress *Tbx5* expression.

### Hoxc9 can repress *Tbx5* expression via Hbs2

**Figure 3:**
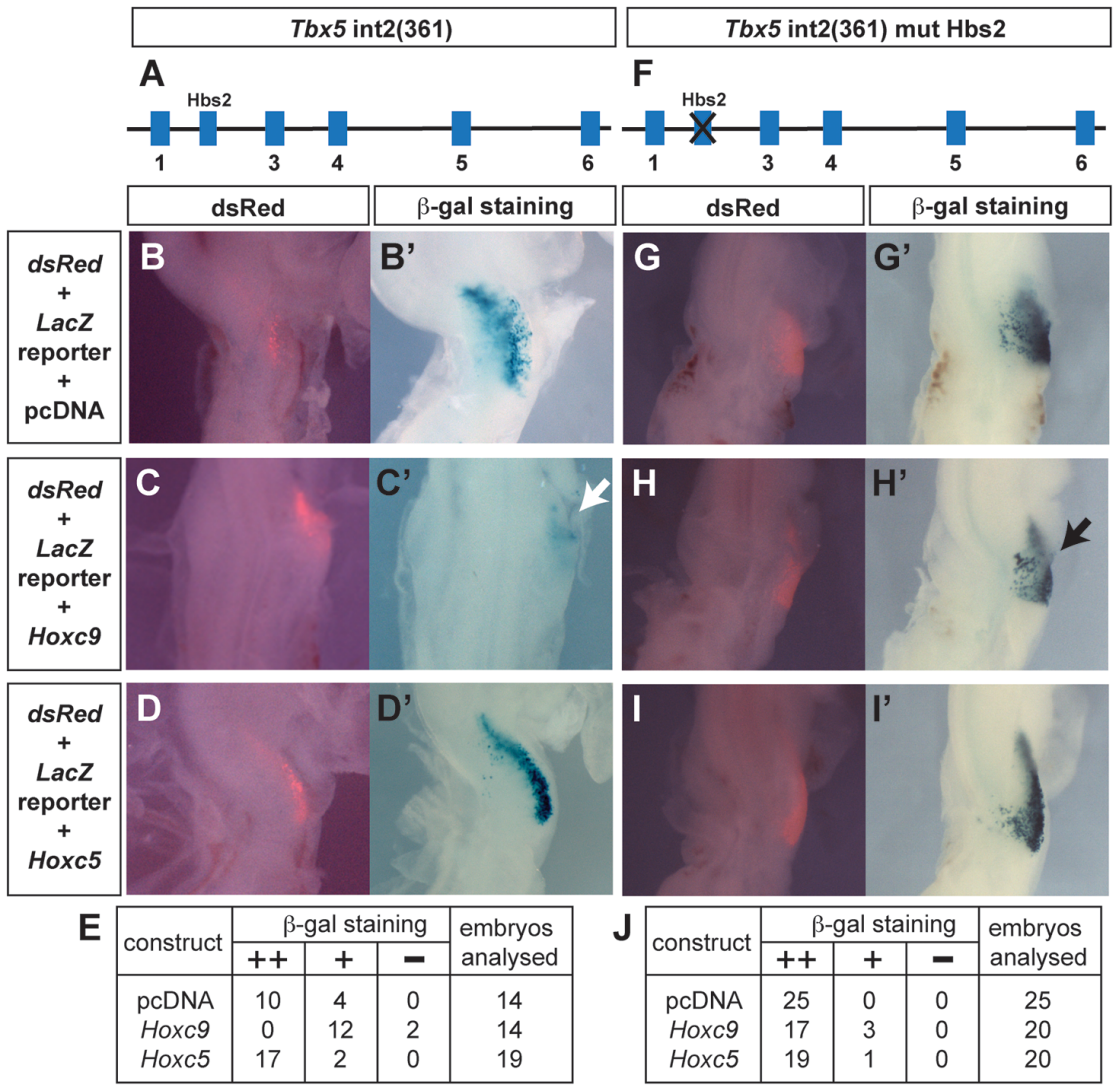
Hoxc9 can repress activity of the *Tbx5* forelimb regulatory element via Hbs2. **A–D.** The *Tbx5* int2(361) *LacZ* reporter construct (A) was electroporated with pCAβ-*dsRed-Express* in the presumptive forelimb region of HH14-15 chick embryos. After 24 hours, electroporation efficiency was assessed by dsRed expression (B–D) and embryos were stained for LacZ to analyze the enhancer activity (B′–D′). Co-electroporation of pcDNA-m*Hoxc9* (C–C′) but not pcDNA-m*Hoxc5* (D–D′) reduced *LacZ* expression. **E.** Tabulation of the numbers of embryos showing β-galactosidase staining for the constructs described in B–D. **F–I.** Equivalent series with the *Tbx5* int2(361) reporter plasmid with mutations on Hbs2 (F). The reporter plasmid was electroporated with pCAβ-*dsRed-Express* and assessed for dsRed and LacZ activity (G–G′). With the Hbs2 mutant reporter, co-electroporation of Hoxc9 (H–H′) did not repress LacZ activity. As in the control, Hoxc5 (I–I′) did not affect enhancer activity. **J.** Tabulation of the numbers of embryos showing β-galactosidase staining for the constructs described in G–I.

To determine whether caudally-expressed *Hox* genes can repress the *Tbx5* forelimb-regulatory element, we compared the activities of *Hoxc9* and *Hoxc5* expression constructs when co-electroporated with the wild type *Tbx5* int2(361) (Fig. 3A) *LacZ* reporter into the forelimb-forming region of HH stage 14–15 chick embryos. As expected, following electroporation of the *Tbx5* int2(361) construct (with a dsRed reporter to assess electroporation efficiency (Fig. 3B)), β-gal activity is detected in successfully targeted forelimb LPM (Fig. 3B′) indicating that this mouse *Tbx5* regulatory element can also function in chick. Following co-electroporation of a *Hoxc9* expression construct with the *Tbx5* int2(361) reporter, *LacZ* expression is repressed in the forelimb region (Fig. 3C′ white arrow). In contrast, performing the equivalent experiment with *Hoxc5*, which is expressed in the rostral, *Tbx5*-expressing LPM, does not negatively effect *LacZ* expression from the reporter (Fig. 3D′) demonstrating that the repressive activity is restricted to caudally-restricted Hox genes, such as *Hoxc9*.

To determine whether Hoxc9 functions via Hbs2 to repress *Tbx5* expression, we co-electroporated *Hoxc9* with a *Tbx5* int2(361) reporter in which Hbs2 is mutated (Fig. 3F). In transgenic mice, this mutation caused *LacZ* expression throughout the forelimb, interlimb and hindlimb regions (Fig. 1N) and electroporation of this reporter alone in the forelimb-forming region produced *LacZ* expression (Fig. 3G′) where cells have been successfully targeted as shown by the dsRed reporter (Fig. 3G). Co-electroporation of *Hoxc9* with the Hbs2 mutated reporter did not repress *LacZ* expression (Fig. 3H′ black arrow). As expected, no effect was observed following co-electroporation with the *Hoxc5* construct (Fig. 3I′). Since the expression of *Hoxc8* and *Hoxc10* are also restricted in caudal LPM, we tested if they can also repress the *Tbx5* reporter activity similar to *Hoxc9*. Ectopic expression of either *Hoxc8* (Fig. S1B′) or *Hoxc10* (Fig. S1C′) reduced *LacZ* expression. Together, these results demonstrate that *Hoxc8/9/10*, which are normally expressed in the caudal LPM, have the ability to repress the *Tbx5* regulatory element and that this repression is mediated via the Hbs2. In contrast, *Hoxc5* does not exhibit equivalent repressive activity.

### Ectopic Hoxc9 can repress *Tbx5* expression

**Figure 4:**
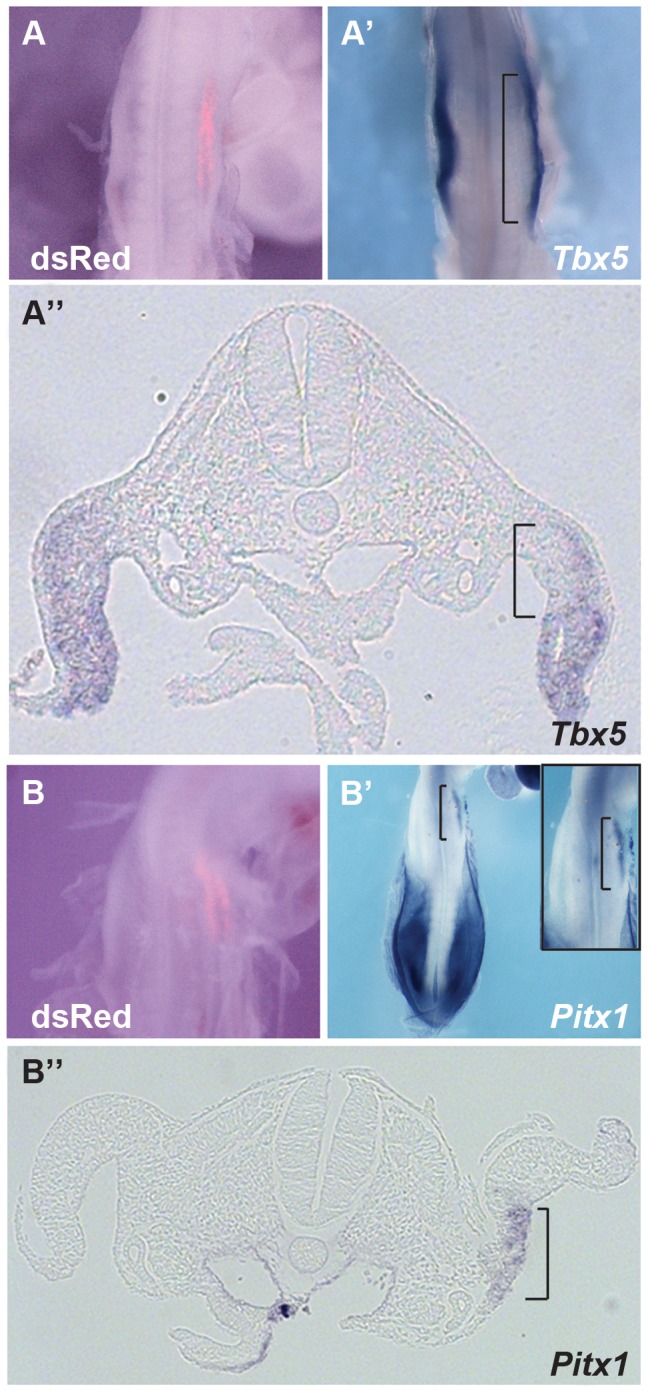
Hoxc9 can repress endogeneous *Tbx5* expression. **A–A″.** pCAGGS-m*Hoxc9* was electroporated and *Tbx5* expression was examined by whole mount *in situ* hybridization. dsRed was used to assay electroporation efficiency(A). *Tbx5* was repressed in the electroporated right forelimb LPM (A′). Section of the embryo shown in panel A and A′ (A″). The region affected is bracketed. **B–B″.** *Pitx1*, a hindlimb-restricted gene, was induced ectopically in forelimb LPM after Hoxc9 ectopic expression (B′). The right panel shows a higher-magnification image. Section of the embryo shown in panel B and B′ (B″). The region affected is bracketed.

We next tested whether ectopic expression of *Hoxc9* could repress endogenous *Tbx5* expression in the forelimb-forming region. Electroporation of the right forelimb-forming region (Fig. 4A) with a *Hoxc9* expression construct can repress the endogenous domain of *Tbx5* (Fig. 4A′–A″). The electroporation protocol targets the proximal LPM most successfully and this is where the most profound repression of *Tbx5* is observed consistent with Hoxc9 acting cell-autonomously.

Although *Tbx5* does not determine forelimb morphologies [13], its forelimb-restricted expression serves as a marker of forelimb identity. Since *Hoxc9* is expressed in caudal LPM including the hindlimb region, we examined if, following ectopic activation of *Hoxc9*, hindlimb markers were activated in the forelimb region concomitant with down-regulation of *Tbx5*. *Pitx1* is expressed in hindlimb, but not in forelimb, and determines some hindlimb morphologies [21]–[24]. Indeed, ectopic *Pitx1* transcripts are detected in the forelimb (Fig. 4B′–B″) following electroporation of a *Hoxc9* expression vector (Fig. 4B). The domain of ectopic *Pitx1* is apparent in the proximal forelimb LPM consistent with the proximal bias in cells successfully targeted by electroporation and again consistent with a cell-autonomous mechanism of action.

### Hox proteins can directly bind to Hbs2

**Figure 5:**
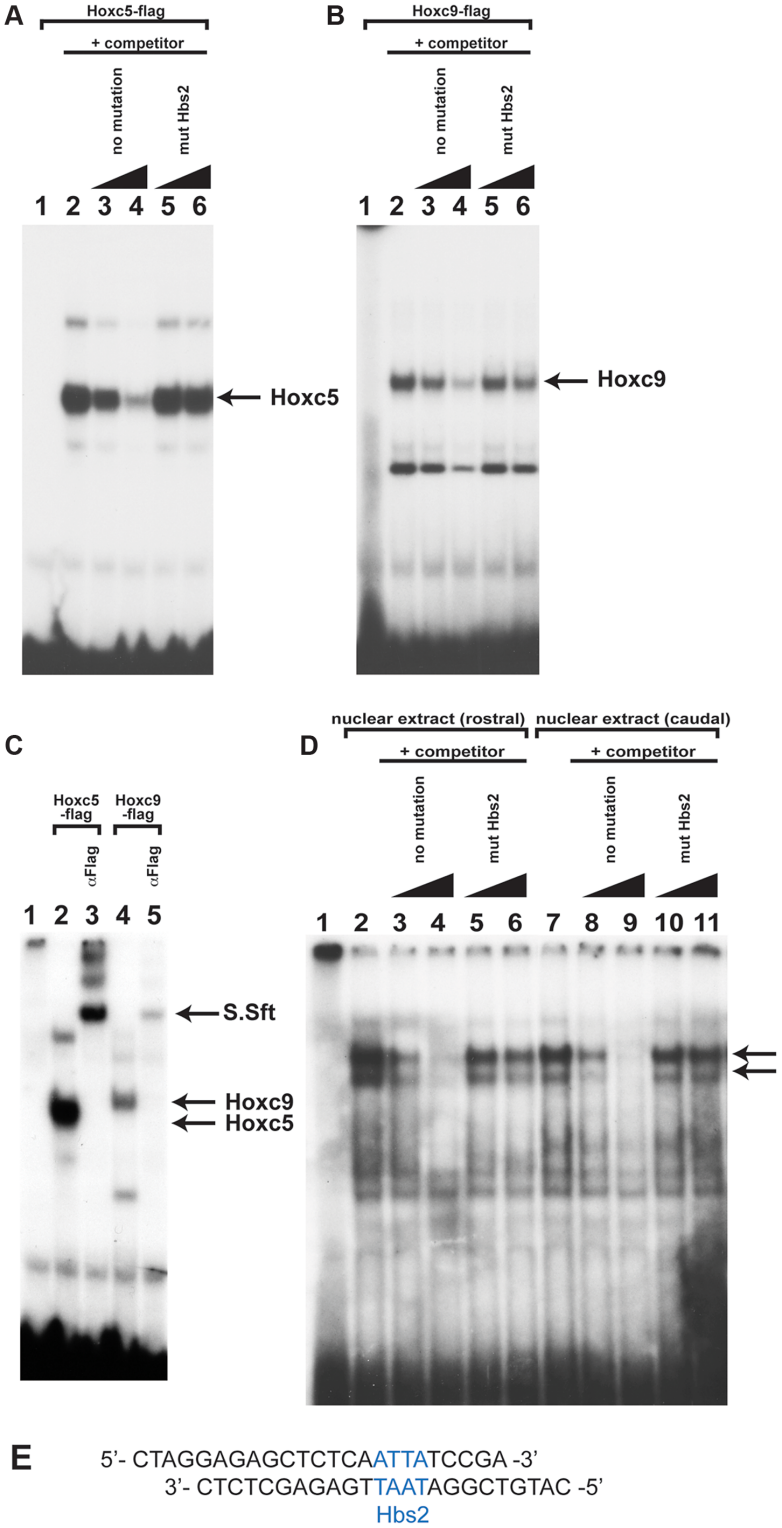
Hox proteins can bind the Hbs2 site. **A.** Binding of *in vitro* translated Hoxc5 flag-tagged proteins to the Hbs2 site. Hoxc5 forms a complex with an oligonucleotide probe containing Hbs2 (lane2) and this can be specifically competed with unlabelled oligo (lane 3–4). Unlabelled oligo containing mutated Hbs2 (mut Hbs2) does not compete with labelled probe (lane 5–6). **B.** Hoxc9 also makes a complex with a probe containing Hbs2 (lane 2) and can be competed with unlabelled probe but not by mutated Hbs2 (mut Hbs2) probe (lane 3–6). **C.** The Hoxc5-Hbs2 complex (lane 2–3) and the Hoxc9-Hbs2 complex (lane 4–5) can be super-shifted by addition of α-flag antibody (lanes 3 and 5). **D.** EMSA using nuclear extracts obtained from forelimb-forming rostral LPM (lane 2–6) or caudal LPM (lane 7–11) of E9.0 mouse embryos. 2 specific bands are produced (arrowed) from both rostral and caudal extracts (lane 2 and 7). Competition assay is performed using no mutation oligo (lane 3–4 and 8–9) or mut Hbs2 oligo (lane 5–6 and 10–11). **E.** Sequence of the oligonucleotide probe used containing Hbs2 (blue).

To understand the molecular mechanisms of caudal Hox-specific repressive activity on *Tbx5* expression, we compared the DNA binding abilities of Hoxc5 and Hoxc9 since paralogous-specific functions of Hox can be explained by different DNA binding specificities [25]. We performed electrophoretic mobility shift assays (EMSA) with an oligonucleotide probe that contains Hbs2 (Fig. 5E). *in vitro* translated Hoxc5 can bind to the probe (Fig. 5A lane 2). Addition of non-labelled oligo as a competitor abolished the DNA-protein complexes showing their specificity (Fig. 5A lane 3–4). Non-labelled oligo in which Hbs2 is mutated (mut Hbs2) did not affect the complexes, confirming that the protein occupies Hbs2 (Fig. 5A lane 5–6). Similar to Hoxc5, Hoxc9 makes a complex with this probe (Fig. 5B lane 2) and the specificity was confirmed by a competition assay (Fig. 5B lane 3–6). We then performed a super-shift assay using an antibody against a flag epitope present in the C- terminal of our recombinant Hox proteins (Fig. 5C). Addition of this antibody resulted in super-shifts of DNA-protein complexes (Fig. 5C lane 3 and 5), indicating these complexes contain Hoxc5 or Hoxc9 proteins. These results suggest that both Hoxc5 and Hoxc9 can bind Hbs2 *in vitro*.

To examine whether the occupancy of Hbs2 in forelimb forming, *Tbx5*-positive LPM and *Tbx5*-negative caudal LPM is different, we carried out EMSA analysis using nuclear extracts from rostral or caudal LPM. We observed two bands of the same size using both rostral and caudal extracts (Fig. 5D lane 2 and 7 arrows). We confirmed the specificity of Hox binding by competition assay. While the no mutation oligo disrupts both of the two bands (Fig. 5D lane 3–4 and lane 8–9) the mut Hbs2 oligo can only very weakly compete the complexes (Fig. 5D lane 5–6 and lane 10–11), suggesting that Hbs2 is required for these DNA-protein complexes.

These results suggest that *in vitro* translated Hoxc9 and Hoxc5 can bind equivalently to Hbs2 and that the protein-DNA complexes from both rostral and caudal nuclear extract occupy Hbs2 specifically. Since the electroporation experiments demonstrate that the repression of the *Tbx5* enhancer by Hoxc9 requires Hbs2 (Fig. 3), one of the Hox proteins forming a complex on Hbs2 using caudal nuclear extract as input is likely to be Hoxc9. In rostral LPM, since the repressive Hox genes, such as Hoxc8/9/10, are not expressed, the Hox proteins on Hbs2 using rostral nuclear extract as input are either activating Hox proteins, such as Hox PG4 and PG5 or Hox proteins with neutral function on *Tbx5* expression. Thus, we propose a model in which HoxPG4 and PG5 protein complexes occupy Hbs2 in rostral forelimb forming LPM, while in *Tbx5*-negative caudal LPM the same site is occupied by Hoxc9 and/or Hoxc8/Hoxc10 containing-complexes that repress *Tbx5* expression. Therefore, we conclude that a combination of restricted expression of Hox genes and the distinct activities of Hox proteins of different paralogous groups, which we demonstrate here, are harnessed to enable restricted expression of *Tbx5* via the Hbs2.

### The N-terminal region of Hoxc9 is sufficient to confer the ability to transcriptionally repress *Tbx5*

**Figure 6:**
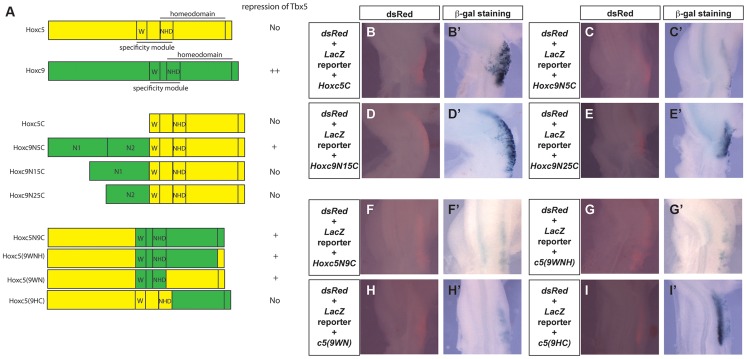
Functional mapping of the Hoxc9 repressor domains. **A.** Schematic representation of Hoxc5, Hoxc9 and chimeric proteins. Domains from Hoxc5 and Hoxc9 are shown in yellow and green, respectively. The specificity module is comprised of a domain including the hexapeptide motif (W) and N-terminal residues of the homeodomain (NHD). **B–I.** The *Tbx5* int2(361) *LacZ* reporter construct was electroporated together with constructs encoding chimeric proteins. dsRed expression (from the pCAβ-*dsRed-Express* reporter) indicating successful targeting of the forelimb 24 hours after electroporation (B–I). LacZ staining of the same embryos shown in B–I (B′–I′). Co-electroporation of a Hoxc5C expression construct has no effect on the activation of the reporter in the forelimb (B′). Reporter expression is repressed by Hoxc9N5C (C′) but not by Hoxc9N15C (D′) or Hoxc9N25C (E′). Reporter expression is repressed by Hoxc5N9C (F′), Hoxc5(9WNH) (G′) and Hoxc5(9WN) (H′) but not by Hoxc5(9HC) (I′).

To further analyse the functional differences between Hoxc5 and Hoxc9, we generated chimeric forms of Hoxc5 and Hoxc9 proteins (Fig. 6A) and assayed their ability to repress the *Tbx5* intron2 reporter construct (Fig. 6B–I). In both Hoxc5 and Hoxc9 the homeodomain is located in the C-terminus of the proteins. Paralog-specific DNA-binding properties have been reported to be determined by a specificity module spanning a Pbx-binding hexapeptide motif (W) present N-terminal to the homeodomain and the N-terminal arm of the homeodomain (NHD) [26] Fig. 6A). As would be predicted, a construct containing only the C-terminal half of Hoxc5 (Hoxc5C) cannot repress reporter gene expression (Fig. 6B–B′). Strikingly, addition of the N-terminal domain of Hoxc9 (N1N2) to the C-terminal half of Hoxc5 converts Hoxc5C into a chimeric protein (Hoxc9N5C) with Hoxc9-like repressor activity (Fig. 6C–C′). This supports our model that the opposing transcriptional activities of Hoxc5 and Hoxc9 do not lie in their distinct ability to bind Hox binding sites. To attempt to further refine the domain(s) responsible for transcriptional repression of *Tbx5*, we divided the N-terminus of Hoxc9 into two smaller domains, Hoxc9N1 and Hoxc9N2, and tested their function. Neither chimeric protein (Hoxc9N15C or Hoxc9N25C) showed clear repression of the reporter demonstrating that within the limits of this assay the entire N-terminus or the domain overlapping the junction between N1 and N2 is required for repressive activity (Fig. 6D–E).

### The specificity module of Hoxc9 can change the transcriptional properties of Hoxc5

Although Hoxc9N5C reduced a reporter gene expression, this repression was weaker than that seen with full length Hoxc9. We, therefore, examined if there are other domains in the C-terminal half of Hoxc9 that can contribute to transcriptional repression. A chimeric protein that contains the N-terminal half of Hoxc5 and C-terminal half of Hoxc9 (Hoxc5N9C) can reduce LacZ expression (Fig. 6F–F′), suggesting that there is an additional repression domain(s) in the C-terminal region of Hoxc9. Replacement of a short C-terminal tail (Hoxc5(9WNH)) with equivalent regions of Hoxc5 did not affect its ability to repress the reporter (Fig. 6G–G′). Strikingly insertion of 18 amino acids spanning the hexapeptides and the homeodomain N-terminal arm from Hoxc9 (Hoxc5(9WN)) is sufficient to convert Hoxc5 to a transcriptional repressor (Fig. 6H–H′). To further test the requirement of these domains, we generated another chimeric protein in which all of the regions upstream from homeodomain N-terminal arm were replaced (Hoxc5(9HC)). This protein did not suppress LacZ expression (Fig. 6I–I′). To confirm that the loss of repressive activity is not caused by the disruption of the 3D-structure of the chimeric protein, we performed EMSA to demonstrate that this protein (Hoxc5(9HC) and the chimeric proteins Hoxc5N9C and Hoxc5(9WNH) can all bind a DNA probe containing Hbs2 (data not shown). These results suggest that repression of *Tbx5* by Hoxc9 is mediated by two domains: one N-terminal and the other in the specificity module that contains Pbx-binding hexapeptides and the N-terminal arm of the homeodomain.

## Discussion

**Figure 7:**
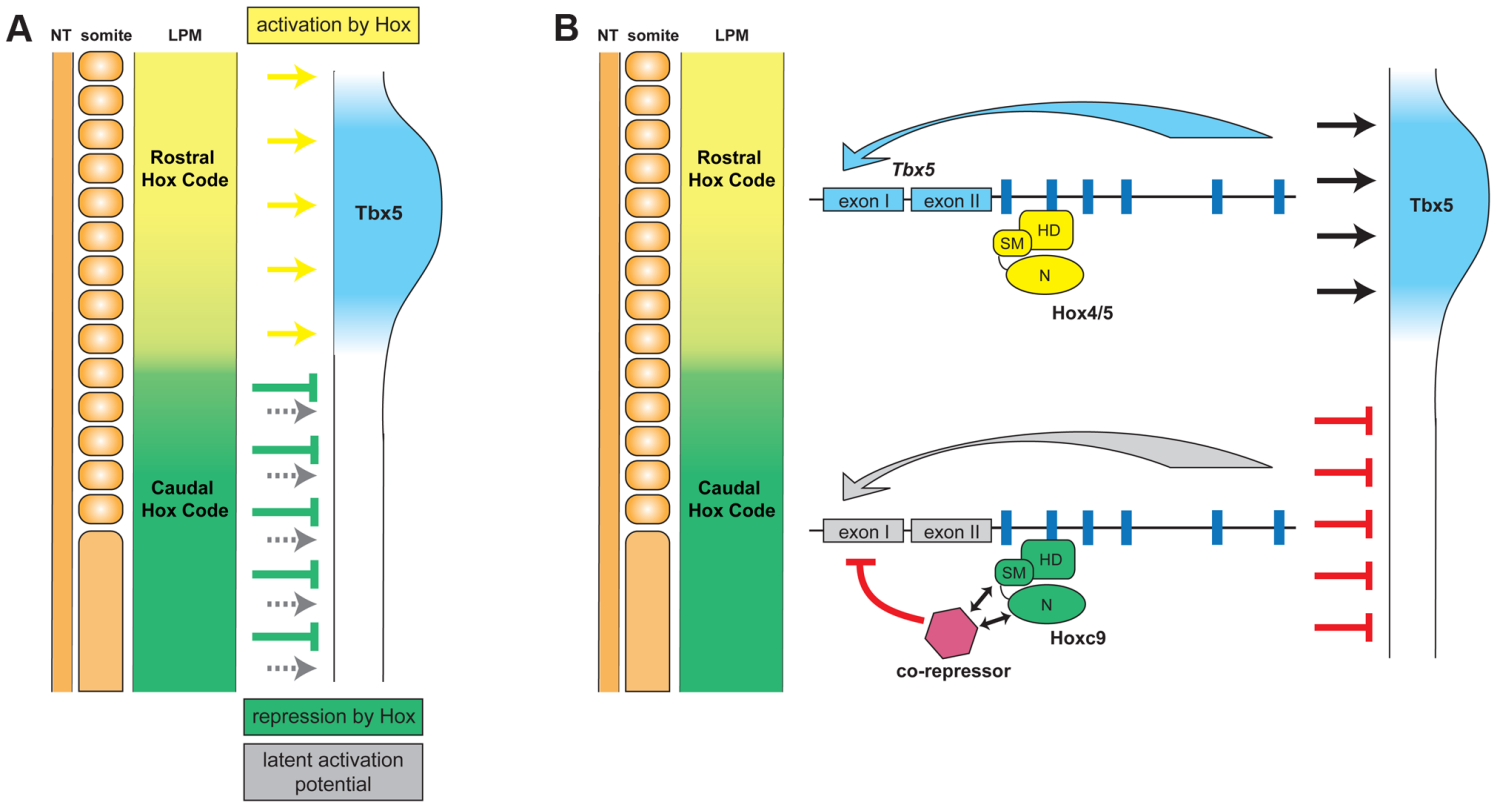
Model for the combinatorial regulation of forelimb-restricted *Tbx5* expression by distinct paralogous Hox gene inputs. **A.** Hox genes expressed in the rostral forelimb-forming LPM induce *Tbx5* expression (yellow arrows). In the caudal flank there is a latent capacity to activate *Tbx5* expression (grey arrows) that is normally masked by the presence of caudally-expressed Hox genes (*Hoxc8*, *Hoxc9* and *Hoxc10*) that repress expression of *Tbx5* (green arrows). Thus, a combination of Hox colinear expression and the specific activator or repressor activities of distinct Hox protein paralogs dictates positioning of forelimb-forming region. **B.** The transcriptional repression of *Tbx5* is controlled by caudally-expressed Hox genes, such as Hoxc9, bound on Hbs2. This site is occupied both in forelimb-forming region (Hox PG4/5) and in caudal LPM (Hoxc9). However, only Hoxc9 forms a repressive complex by recruiting co-repressor(s). HD, homeodomain; SM, specificity module; N, N-terminus.

Using the forelimb regulatory element of *Tbx5* as an assay, we have been able to distinguish the opposing transcriptional activities of different Hox paralogous group proteins. Hoxc9, as well as Hoxc8 and Hoxc10, that are normally expressed in the LPM caudal to the forelimb, can repress *Tbx5* to restrict its expression to the forelimb level region of the LPM (Fig. 7). A single Hox binding site (Hbs2) in the *Tbx5* forelimb enhancer is required for this restriction through repression, as mutation of this site causes caudal expansion of expression. Hoxc9 can suppress *Tbx5* transcription through this site and this repressive activity is restricted to caudal Hox proteins. Previously, we showed that Hox4 and Hox5 paralogs positively regulate *Tbx5* expression [19]. We reveal the combinatorial regulation of *Tbx5* by distinct paralogous Hox gene inputs. Hox PG4 and PG5 genes expressed in forelimb-forming LPM form a transcriptional activation complex to positively regulate *Tbx5*, while Hoxc9, as well as Hoxc8 and Hoxc10 genes expressed in LPM at more caudal levels form a repressive complex to restrict *Tbx5* expression. Together, our results reveal that the forelimb-restricted expression of *Tbx5* is achieved through co-option of two characteristics of Hox genes, their colinear expression pattern along the rostro-caudal body axis and the functional specificity of Hox proteins from different paralogous groups.

### Hox binding site specificity

Our results demonstrate that five of the six Hox binding sites (namely Hbs1 and Hbs3-6) within the *Tbx5* forelimb regulatory element are required for the positive regulation of *Tbx5* expression while a single site (Hbs2) is required for its repression in caudal LPM (Fig. 1). One possible mechanism to explain how these opposing transcriptional effects are mediated is that different Hox proteins have distinct binding preferences for these sites. For example Hox proteins that act as activators, such as Hox PG4 and PG5, have greater affinity for Hbs1 and 3-6 while repressive Hox proteins, such as Hoxc8/9/10, preferentially bind Hbs2. Our results do not support such a model. In this study, we show that both Hoxc5 and Hoxc9 proteins can bind repressive Hbs2 sites (Fig. 5), suggesting the repressive activity of Hbs2 is not mediated by preferential binding of repressive Hox proteins.

An alternative model is that the transcriptional activity of the Hox complex bound at Hbs2 is determined by a co-factor(s). The sequence of Hbs2 is identical to the sequences of Hbs1 and Hbs3, therefore we compared the sequences surrounding these Hox binding sites. One distinguishing feature of Hbs2 identified using Mat Inspector (http://www.genomatix.de) is the presence of a 6-bp sequence named Pbx1-Meis1 complexes site located 3′ of Hbs2 (6bp3′). Pbx is a Hox co-factor that can attenuate Hox-mediated gene transcription by recruiting histone deacetylases (HDACs) [27]. Therefore, a possible mechanism of the transcriptional repression through Hbs2 is the recruitment of HDACs to Hbs2/6bp3′ by Pbx. To examine this model, we mutated this 6 bp sequence while leaving Hbs2 intact. This mutation did not cause expansion of the reporter gene unlike mutation of Hbs2 or mutations of both Hbs2 and 6bp3′ (Fig. 1), suggesting that the repression is independent of 6bp3′. Thus, our results do not support a role for Pbx determining the transcriptional activities of Hox proteins bound to the *Tbx5* forelimb regulatory element.

### Specificity of Hox function

The specificities of Hox proteins from different paralogous groups must be tightly regulated. One mechanism by which this is achieved is through distinct DNA binding specificity, for example homeodomains of Hoxc5 and Hoxc9 have different sequence preference in protein binding microarrays [25]. We found, however, that both Hoxc5 and Hoxc9 can bind Hbs2 (Fig. 5), suggesting specificity is not determined by distinct DNA binding abilities of Hox proteins. In addition, we also demonstrate that Hoxc9N5C chimeric protein, which contains the N-terminal repression domain of Hoxc9 fused to the homeodomain –containing C-terminus of Hoxc5, can repress *Tbx5*. Thus, the transcriptional repression specific to Hoxc9 is not mediated by DNA-binding specificity but rather achieved by transcriptional repression activities restricted to Hoxc9, which are mediated by two domains; the specificity module including the Pbx-binding hexapeptide and homeodomain N-terminal arm and a region N-terminal to the specificity module (Fig. 7).

The mechanism by which these domains confer repressive activity remains to be elucidated. One possible model is by interacting with other transcriptional regulatory domain(s) in the protein. The hexapeptide of AbdA represses dpp expression by inhibiting the function of a glutamine (Q)-rich C-terminal activation domain [28]. Mutations in the hexapeptide converts AbdA from a repressor to an activator without affecting DNA-binding site selection. Although Hoxc9 lacks this Q-rich domain, the hexapeptide of Hoxc9 may block the activity of an unidentified activation domain. Another possibility is that the length of the linker region between the hexapeptide and homeodomain determines transcriptional activity. Several Antp isoforms are produced that have different linker sizes. Synthetic Antp protein with a long linker behaves as an activator, while the short-linker construct acts as a repressor, suggesting the importance of linker size [29]. As Hoxc9 has a shorter linker than Hoxc5, this may favour its function as a repressor.

As it is unlikely that Hox protein itself directly represses *Tbx5* transcription, we suggest the model that Hoxc9 supresses *Tbx5* expression by interaction with co-repressor(s) (Fig. 7). One candidate is histone deacetylase (HDAC), which can bind Hox proteins directly [30], however, in EMSA we were unable to detect a HDAC/Hoxc9 complex on Hbs2, with *in vitro* translated proteins or nuclear extract from LPM (data not shown). Other potential collaborators are Smad proteins. In the *Drosophila* haltere, a Mad/Med/Shn complex works in combination with Ubx to repress *Sal* expression [31]. There is a potential Smad binding site proximal to Hbs2, however, we mutated this site and did not observe expansion in expression, rather it caused reduced expression in the distal limb bud, suggesting this Smad binding site may have a positive input on *Tbx5* expression (). Other candidate repressors are engrailed (En) and sloppy paired (Slp) since, in *Drosophila*, they form a complex with Hox, Exd and Hth to repress transcription [32]–[34]. Neither of the two mouse En genes, *Engrailed1* and *Engrailed2*, are expressed in LPM at pre-limb bud stages [35], [36]. The mammalian homolog of Slp, fork head box G1 (FoxG1)/brain factor 1 (BF-1) is also not expressed in LPM [37]. Therefore, the putative co-repressors enabling unique Hoxc9 repressive activity remain to be determined.

We have shown that Hoxc8 and Hoxc10 have transcriptional repression ability similar to Hoxc9 (Fig. S1). To gain an insight of the mechanisms of their function, we compared the amino acid sequences of Hoxc8, Hoxc9 and Hoxc10 (data not shown). We could not, however, find any obvious conserved domains outside of homeodomains. It is possible that they use different mechanisms to repress *Tbx5* expression or that they share similar 3D structure domains in spite of their distinct amino acid sequences.

### Patterning of LPM

Our analysis of the *Tbx5* forelimb regulatory element reveals a direct link between patterning of the rostro-caudal axis of the embryo by Hox genes and the programme that controls positioning of the forelimb forming territory. A clear correlation between Hox expression and establishment of the forelimb territory of the LPM has previously been suggested [17], [18], [38]. Application of Fgf to the interlimb flank adjacent to the normal wing induces a wing-like extra limb that expresses *Tbx5* [5], [39]. Prior to the emergence of the ectopic wing the endogenous expression of *Hoxc9* is reduced [38] consistent with downregulation of Hoxc9 as a repressor of *Tbx5* (and the subsequent forelimb programme) being essential for emergence of an ectopic wing bud from this region. In the limbless python, Hoxc8 expression is rostrally expanded to the anterior limit of the trunk [18]. Hoxc8 is expressed exclusively in *Tbx5*-negative caudal LPM at pre-limb bud stages in chick and mouse (Fig. 2) and it can, like Hoxc9, repress *Tbx5* (Fig. S1). Our results therefore, explain the mechanisms that lead to loss of forelimbs in snake through the repression of *Tbx5* following expansion of Hoxc8 expression throughout the trunk.

A previous study has demonstrated the presence of and a function for *Hox9* genes in anterior-posterior patterning of the forelimb [40]. The complete loss of *Hox9* paralogous group leads to the loss of *Hand2* expression in posterior forelimb and a consequent reduction in *Shh* expression, while no effect on *Tbx5* expression was reported. Failure to observe any caudal expansion of *Tbx5* in this mutant can be simply explained by the redundant function of Hoxc8 and Hoxc10. The same study reported that *Hoxc9* is expressed in the forelimb bud at E9.5, but it is undetectable by E10.5. *Tbx5* expression is first initiated in the forelimb-forming region at E8.5. We therefore examined the expression of *Hoxc9* at stages E8.5–E9.5 (data not shown), however, we did not detect expression of *Hoxc9* in the forelimb-forming region, in contrast to the strong staining in caudal tissues. We therefore conclude that Hoxc9 is not present in the forelimb-forming region at stages when *Tbx5* expression is first initiated. Later expression of *Hoxc9* is not sufficient to cause detectable repression of the domain of *Tbx5* already activated by Hox4/5 paralogous genes.

While we have shown that Hoxc8, Hoxc9 and Hoxc10 can repress *Tbx5* expression, our study does not exclude the possibility that other caudally-expressed Hox genes have a similar repressive ability. We favour a model in which other caudally-expressed Hox paralogs have redundant functions in repression of *Tbx5*. *Hoxc* cluster null mice have no defects in the limb skeleton [41], however, the expression of *Tbx5* in these mutants have not been reported and we predict that the ectopic expansion of *Tbx5* in caudal LPM would not cause any skeletal defects. Further analysis will be required to uncover the requirement of caudally–restricted Hox paralogs, such as Hox8, Hox9 and Hox10 for *Tbx5* repression in caudal LPM.

In addition, while our results clearly demonstrate the importance of specific Hox inputs to generate the restricted expression of *Tbx5* in the LPM, a similar Hox protein code is present in axial tissues (neural tube and somites) that do not express *Tbx5*. The activity of the forelimb regulatory element of *Tbx5* is restricted to LPM and this LPM restriction is maintained following mutation of Hbs2 that leads to caudal expansion in expression. One possible explanation for LPM restriction is the presence of unknown repressors in axial tissues or alternatively additional factors, which are active exclusively in LPM, are required for *Tbx5* expression. Odd-skipped related (*Osr*) genes are candidates as they are expressed in LPM, but excluded from axial tissues such as neural tube and somites [42]. We mutated a putative Osr binding site within the *Tbx5* forelimb regulatory element to test if reporter activity was lost. The activity of the element was unaffected, however, suggesting *Osr* genes are not required for *Tbx5* LPM expression (Fig. S3).

### Conclusions

Our analysis of the *Tbx5* forelimb regulatory element has revealed a mechanism by which Hox genes regulate embryonic patterning and how recruitment of regulatory elements allow for the acquisition of novel structures and independent modulation of their morphology. Mechanisms that control PG-specific Hox functions have been described in Drosophila [26], [43]–[48]. Vertebrates, however, have a minimum of 2–4 Hox genes from the same PG and functional redundancy between Hox proteins from the same PG makes it difficult to examine their specific functions experimentally. Here we used a direct target of Hox activity, a regulatory element of *Tbx5*, to analyse the mechanism of Hox functional specificity and distinguished DNA binding specificity and transcriptional activity. Interestingly, the *Tbx5* forelimb regulatory element contains both activating sites and a repressive site in a relatively short fragment of 361 bp. Active complexes are not spatially restricted and can be formed by a range of Hox PG proteins present throughout the rostral-caudal LPM. Instead, restriction of *Tbx5* expression is achieved by superimposing a dominant repressive (Hoxc8, c9 and c10) complex that ultimately determines the caudal boundary of *Tbx5* expression. Thus, the regulation of *Tbx5* expression in the LPM represents an excellent system to understand the interactions between neighbouring Hox binding sites and how the consequent output is integrated.

## Materials and Methods

### DNA constructs and transient transgenic analysis

For reporter analysis in chick and mouse, we used the BGZA reporter vector [49]. Putative DNA binding sites were searched by MatInspector (http://www.genomatix.de). Transgenic embryos were generated by the Procedural Service section, NIMR by standard pronuclear microinjection techniques. Mouse embryos were staged according to [50]. Noon on the day a vaginal plug was observed was taken to be E0.5 days of development. Mice carrying the *LacZ* transgene were identified by PCR using specific primers (*LacZfwd*, 5′GGTCGGCTTACGGCGGTGATTT3′; *LacZrev*, 5′AGCGGCGTCAGCAGTTGTTTTT3′). Sequences surrounding putative Hox binding sites and the mutations induced are as followings, binding sites are shown in bold; Hbs1, AC**ATTA**TTGGA ; mut Hbs1, AC**ATGC**TTGGA; Hbs2, GACTCTCA**ATTA**TC; mut Hbs2, GACTCTCA**ACGA**TC; mut 6bp3′, GACTGCAA**ATTA**TC; mut Hbs2+6bp3′, GACGCTTA**ACGA**TC; Hbs3, AGA**TAAT**TC; mut Hbs3, AGA**TCGT**TC; Hbs4, CC**TTATTA**AGG; mut Hbs4, CC**TTGGCA**AGG; Hbs5, CCAT**TTAT**CTTG; mut Hbs5, CCAT**TCGT**CTTG; Hbs6, TG**TTAT**TT; mut Hbs6, TG**TCGT**TT.

### Whole mount *in situ* hybridization

Whole mount *in situ* hybridizations were carried out essentially as previously described [51]. Probe templates for chick Hox genes, *Pitx1*, *Tbx5* and mouse Hox genes have been described previously [4], [17], [19], [52], [53] Embryos were sectioned by the Histology service, NIMR.

### *In ovo* electroporation of chick embryos

Fertilized chick embryos (Henry Stewart Ltd, Winter Egg Farm) were incubated at 38°C and staged according to Hamburger Hamilton (HH) [54]. Reporter constructs and/or Hox expression constructs were mixed with fast green dye tracer and injected into the coelom located between the somatic and splanchnic LPM. Electric pulses (three pulses 30 v, 50 ms, with 200 ms intervals for tungsten electrodes or three pulses 20 v, 50 ms, with 200 ms intervals for platinum electrodes) were then immediately applied. Only those embryos showing robust expression of dsRed reporter (pCAβ-dsRed-Express) were processed for further analysis.

### *In vitro* translated protein and nuclear extracts from mouse embryos

*In vitro* translated proteins were produced using a TnT Coupled Reticulocyte Lysate System (Promega). Proteins were labelled with 35^S^-Methionine (PerkinElmer) to verify and quantify translation. LPM strips adjacent to somites 5–10 (rostral LPM nuclear extract) and lateral to somite 14 to its caudal extreme (caudal LPM nuclear extract) were dissected from E9 mouse embryos. Nuclear extracts were prepared using the NE-PER Nuclear and Cytoplasmic Extraction Kit (Pierce) following manufacturers instructions.

### Electrophoretic mobility shift assays

Double-strand oligonucleotides were labelled with ^32^P by incubating with T4 polynucleotide kinase (NEB) for 30 minutes. 2 µl of *in vitro* translated protein or nuclear extract were blocked with 200 ng poly-dIdC, 2 µg of poly-dGdC or 2 µg of poly-dAdT in binding buffer (6.7 mM Tris-HCl pH 7.5, 50 mM NaCl, 0.67 mM EDTA, 0.67 mM DTT, 2 µg BSA, 4% glycerol) in a total volume of 22 µl for 15 minutes on ice. For super-shift, 2 µl of the antibody recognising flag epitope (Sigma, F3165) was added to the binding reaction and incubated for a further 15 minutes. Then, 1 µl of ^32^P -labelled double-stranded oligonucleotides were mixed and incubated for 30 minutes. The protein∶DNA hybrids were resolved on 6% PAGE in 0.5xTBE.

## Supporting Information

Figure S1 Hoxc8 and Hoxc10 can repress activity of the *Tbx5* forelimb regulatory element. **A–C.** The *Tbx5* int2(361) *LacZ* reporter construct was electroporated with pCAβ-*dsRed-Express* in the presumptive forelimb region of HH14 chick embryos. 24 hours later, the efficiency of electroporation was assessed by dsRed expression (A–C) and embryos were stained for LacZ to analyze the enhancer activity (A′–C′). Co-electroporation of pcDNA-m*Hoxc8* (B and B′) and pcDNA-m*Hoxc10* (C and C′) reduced LacZ expression.
(TIF)

Figure S2 Disruption of a putative smad binding site does not expand *LacZ* reporter expression. **A,** Schematic representation of the *Tbx5* intron2 regulatory element. A putative smad binding site and Hox binding sites are shown as green and blue boxes, respectively. **B–C,** E9.5 embryos were stained for β-galactosidase for a mutated construct of *Tbx5* int2(565) reporter.
(TIF)

Figure S3 Disruption of a putative Osr binding site does not affect *LacZ* reporter expression. **A,** Schematic representation of the *Tbx5* intron2 regulatory element. A putative Osr binding site (purple box) locates proximal to Hbs5 (blue box). **B–C,** E9.5 embryos were stained for β-galactosidase for mutated constructs of *Tbx5* int2(565) reporter.
(TIF)
