# Supplementary figures and images for: A Combination of Activation and Repression by a Colinear Hox Code Controls Forelimb-Restricted Expression of Tbx5 and Reveals Hox Protein Specificity
Source: PLoS Genet. 2014 Mar 20;10(3):e1004245. doi: 10.1371/journal.pgen.1004245 (PMC3961185; doi:10.1371/journal.pgen.1004245)

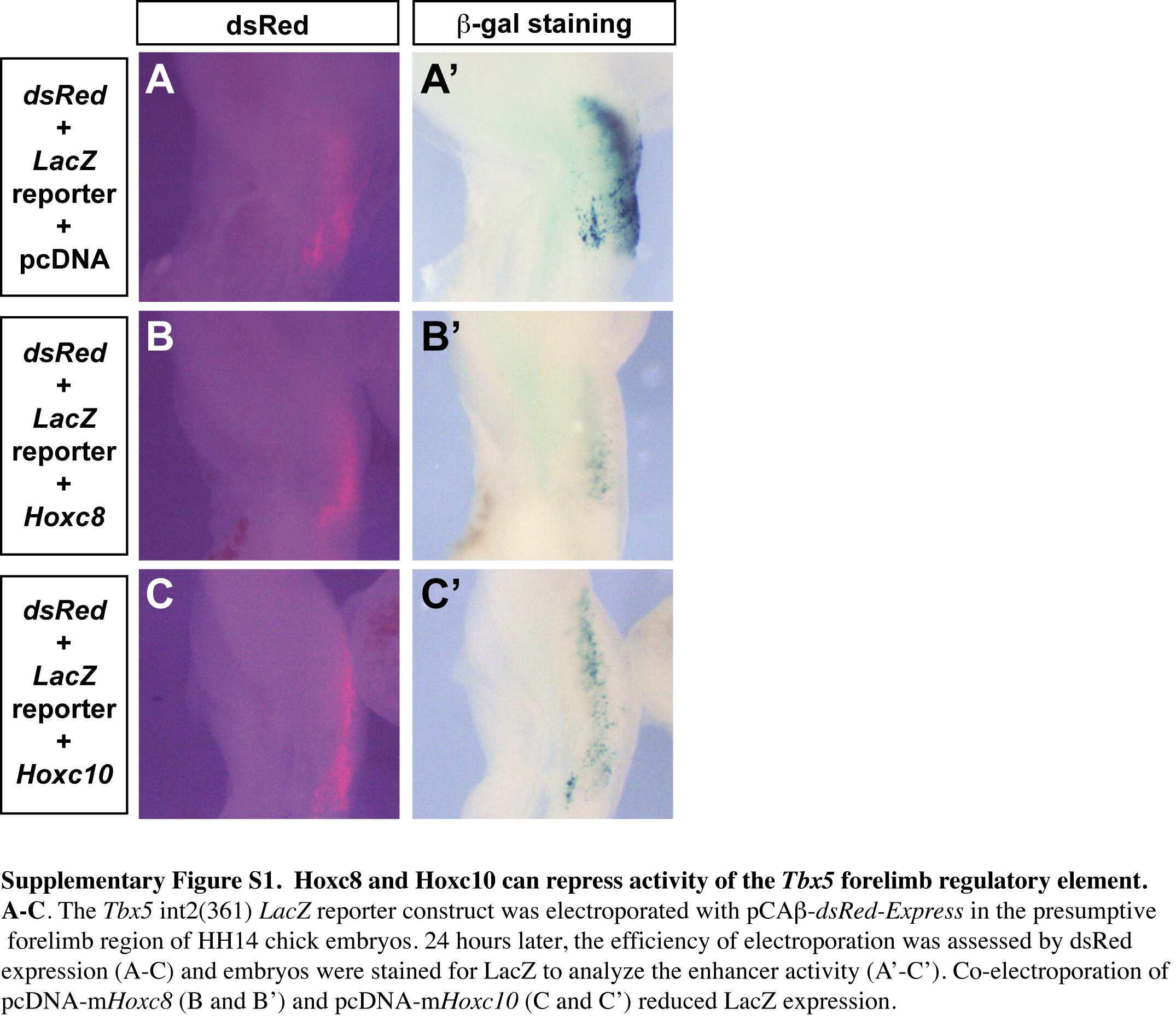

Supplement: Figure S1 — Hoxc8 and Hoxc10 can repress activity of the Tbx5 forelimb regulatory element. A–C. The Tbx5 int2(361) LacZ reporter construct was electroporated with pCAβ-dsRed-Express in the presumptive forelimb region of HH14 chick embryos. 24 hours later, the efficiency of electroporation was assessed by dsRed expression (A–C) and embryos were stained for LacZ to analyze the enhancer activity (A′–C′). Co-electroporation of pcDNA-mHoxc8 (B and B′) and pcDNA-mHoxc10 (C and C′) reduced LacZ expression. (TIF) [file pgen.1004245.s001.tif]

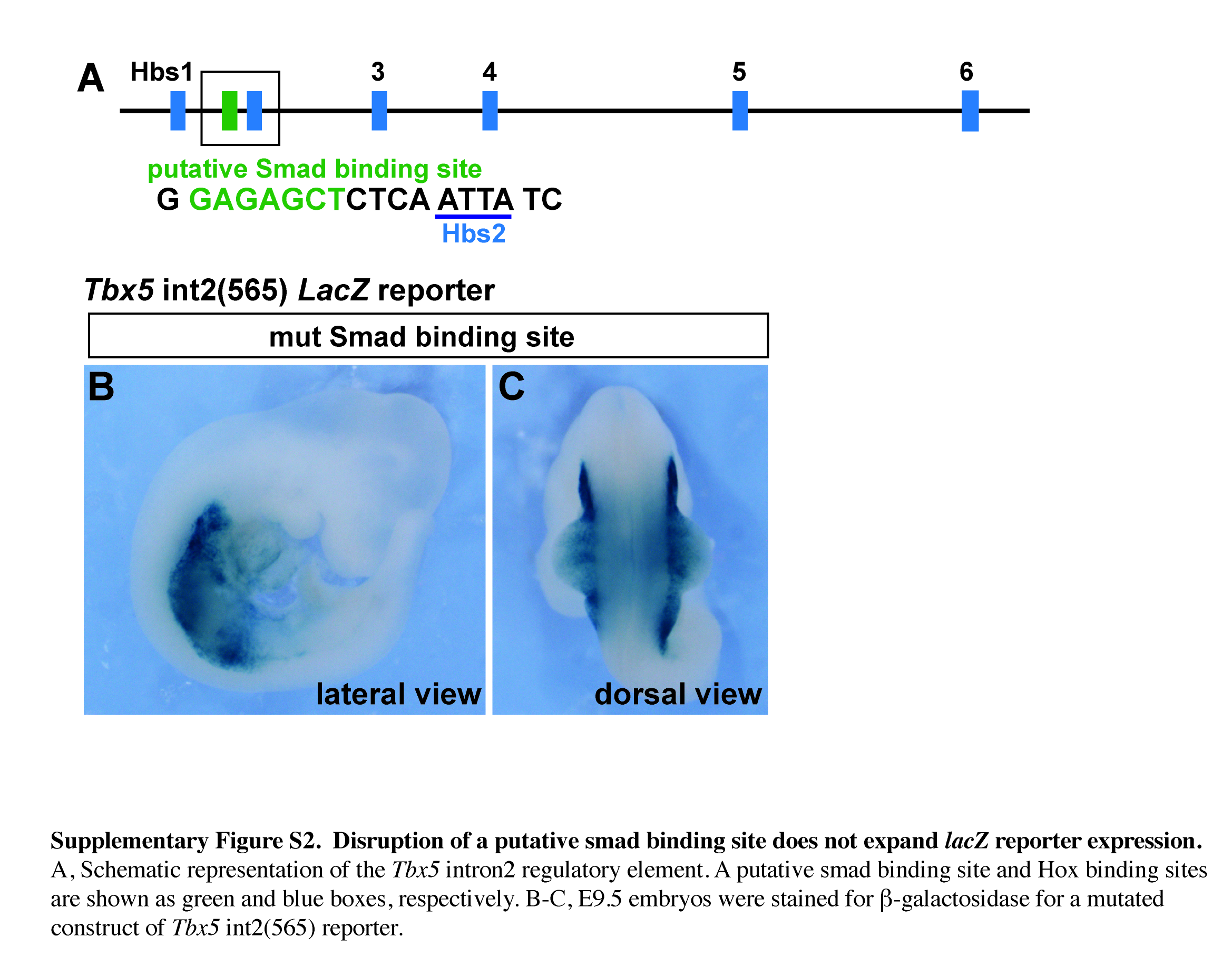

Supplement: Figure S2 — Disruption of a putative smad binding site does not expand LacZ reporter expression. A, Schematic representation of the Tbx5 intron2 regulatory element. A putative smad binding site and Hox binding sites are shown as green and blue boxes, respectively. B–C, E9.5 embryos were stained for β-galactosidase for a mutated construct of Tbx5 int2(565) reporter. (TIF) [file pgen.1004245.s002.tif]

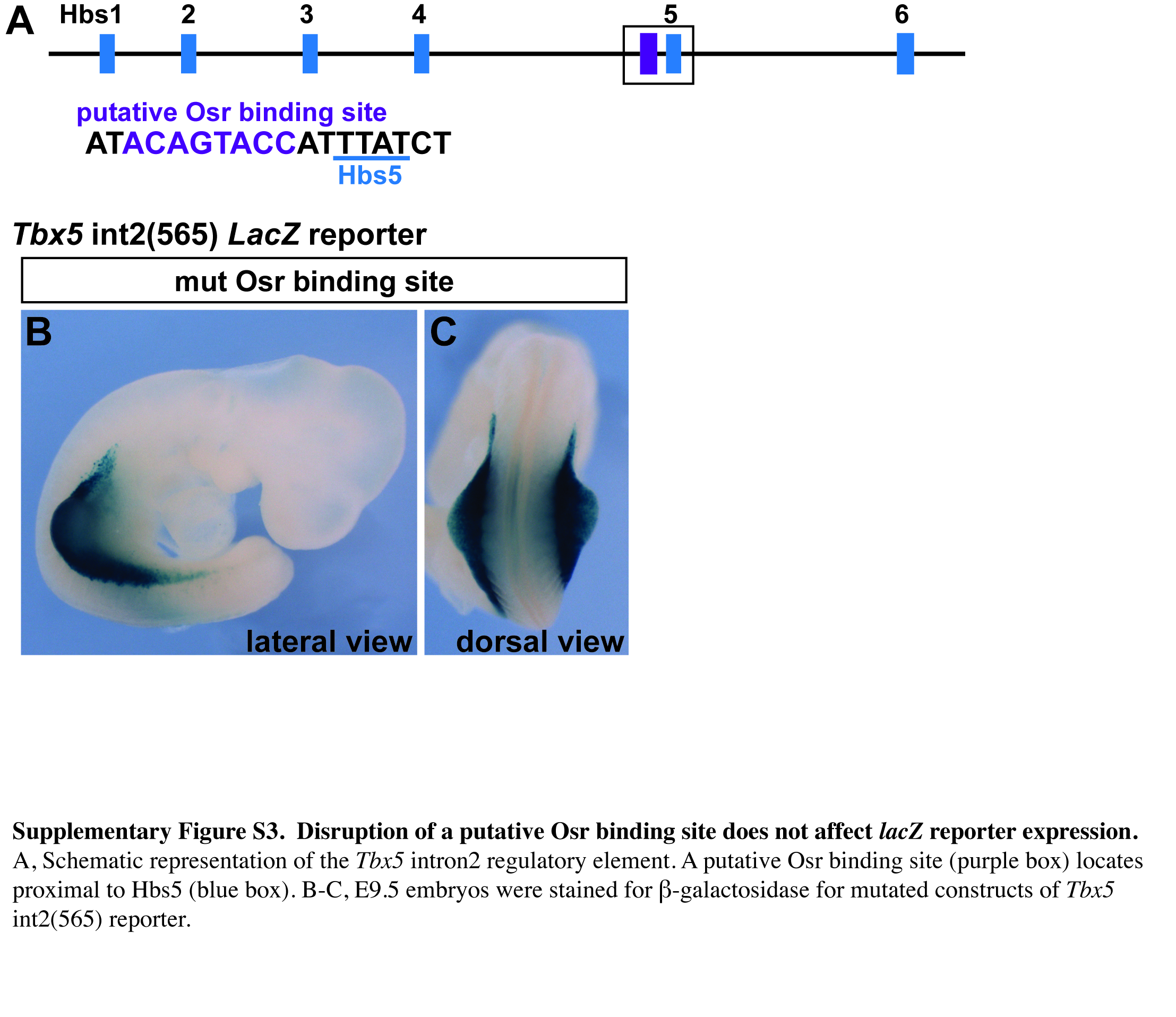

Supplement: Figure S3 — Disruption of a putative Osr binding site does not affect LacZ reporter expression. A, Schematic representation of the Tbx5 intron2 regulatory element. A putative Osr binding site (purple box) locates proximal to Hbs5 (blue box). B–C, E9.5 embryos were stained for β-galactosidase for mutated constructs of Tbx5 int2(565) reporter. (TIF) [file pgen.1004245.s003.tif]
